# Fibroblast-loaded carboxymethyl chitosan—aldehyde hyaluronic acid injectable hydrogel for scleral remodelling to prevent development of myopia

**DOI:** 10.1093/rb/rbaf096

**Published:** 2025-10-10

**Authors:** Jingwen Hui, Kexin Tang, Yuejun Zhou, Ziming Wang, Qian Zhang, Guoge Han, Wenguang Liu, Xiongfeng Nie, Quanhong Han, Xiaoyong Yuan

**Affiliations:** Clinical College of Ophthalmology, Tianjin Medical University, Tianjin 300070, China; Tianjin Eye Hospital, Tianjin Key Laboratory of Ophthalmology and Visual Science, Tianjin Eye Institute, Tianjin 300020, China; Clinical College of Ophthalmology, Tianjin Medical University, Tianjin 300070, China; Tianjin Eye Hospital, Tianjin Key Laboratory of Ophthalmology and Visual Science, Tianjin Eye Institute, Tianjin 300020, China; Clinical College of Ophthalmology, Tianjin Medical University, Tianjin 300070, China; Tianjin Eye Hospital, Tianjin Key Laboratory of Ophthalmology and Visual Science, Tianjin Eye Institute, Tianjin 300020, China; School of Medicine, Nankai University, Tianjin 300350, China; Clinical College of Ophthalmology, Tianjin Medical University, Tianjin 300070, China; Tianjin Eye Hospital, Tianjin Key Laboratory of Ophthalmology and Visual Science, Tianjin Eye Institute, Tianjin 300020, China; School of Material Science and Engineering, Tianjin Key Laboratory of Composite and Functional Materials, Tianjin University, Tianjin 300350, China; Clinical College of Ophthalmology, Tianjin Medical University, Tianjin 300070, China; Tianjin Eye Hospital, Tianjin Key Laboratory of Ophthalmology and Visual Science, Tianjin Eye Institute, Tianjin 300020, China; School of Material Science and Engineering, Tianjin Key Laboratory of Composite and Functional Materials, Tianjin University, Tianjin 300350, China; School of Material Science and Engineering, Tianjin Key Laboratory of Composite and Functional Materials, Tianjin University, Tianjin 300350, China; Clinical College of Ophthalmology, Tianjin Medical University, Tianjin 300070, China; Tianjin Eye Hospital, Tianjin Key Laboratory of Ophthalmology and Visual Science, Tianjin Eye Institute, Tianjin 300020, China; Clinical College of Ophthalmology, Tianjin Medical University, Tianjin 300070, China; Tianjin Eye Hospital, Tianjin Key Laboratory of Ophthalmology and Visual Science, Tianjin Eye Institute, Tianjin 300020, China

**Keywords:** myopia, fibroblast, injectable hydrogel, scleral remodelling

## Abstract

High myopia severely threatens the visual health of adolescents, with pathological features of decreased collagen aggregation and scleral thinning, ultimately leading to axial elongation on preretinal imaging. Fibroblasts play crucial roles in scleral remodelling and myopia prevention. In this work, we developed a fibroblast-loaded carboxymethyl chitosan-aldehyde hyaluronic acid (CMCS-HA-CHO) injectable hydrogel for anti-scleral remodelling. The CMCS-HA-CHO hydrogel is formed through simple mixing under mild conditions via a Schiff base reaction between CMCS and HA-CHO. The CMCS-HA-CHO hydrogel can be injected into a posterior sclera with a low modulus, and the increasing modulus over time provides good mechanical support to the sclera. The hydrogel demonstrated excellent cytocompatibility and haemocompatibility, and the encapsulated fibroblasts maintained good activity. Both the hydrogel and fibroblast-loaded hydrogel effectively shortened the axial length of myopic eyes in guinea pigs in a deprivation model. In particular, the fibroblast-loaded hydrogel had the best therapeutic effect because of the synergy of cell therapy and mechanical support, which always shortened the eye axis within 4 weeks. Furthermore, increased collagen secretion promoted by fibroblasts can increase the thickness of the sclera and improve its biomechanical properties, ultimately repairing the physiological structure of the sclera. This fibroblast-loaded injectable hydrogel may represent a promising clinical approach for controlling myopia progression.

## Graphical abstract

**Figure:**
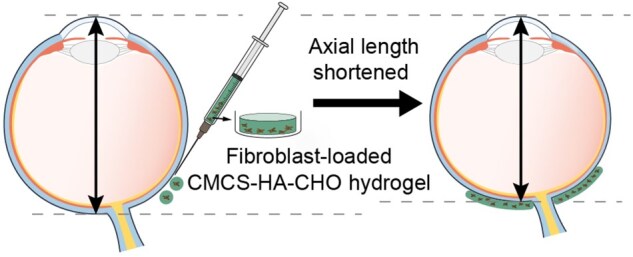

## Introduction

Myopia, particularly pathological myopia, is one of the leading causes of irreversible vision impairment in young and middle-aged populations worldwide [1]. It is typically characterized by pathological changes in the ocular structure as the refractive error and axial length increase [2, 3]. These changes include posterior staphyloma, diffuse atrophy of the retina in the macular region, and degenerative changes such as peripapillary chorioretinal atrophy, which can lead to decreased vision or even blindness [4]. The progression of myopia is associated with decreased scleral collagen aggregation, scleral thinning, and loss of scleral texture, as observed in both human and animal models [5]. Additionally, a reduced diameter of collagen fibrils is noted in the sclera of eyes with pathological myopia [6]. However, the mechanisms underlying the continuous progression of this disease remain unclear, highlighting the importance of early myopia prevention and timely correction.

Posterior scleral reinforcement (PSR) surgery, a surgical procedure aimed at physically reinforcing the posterior segment of the sclera to reduce axial elongation, involves the placement of reinforcing material outside the posterior pole of the eye to provide structural support and has become an effective treatment option to prevent the progression of pathological myopia [7]. PSR can effectively slow or even halt the progression of pathological myopia by providing mechanical support to the thinning and elongating sclera, potentially preventing severe myopic complications, such as retinal detachment, myopic maculopathy, and glaucoma [8]. By limiting axial elongation of the eye, PSR helps stabilize visual acuity and reduces the risk of further deterioration [9], which is particularly beneficial for patients with rapidly progressing myopia [10]. However, owing to the current limits of PSR, its success can vary according to the surgical capability of different doctors. Moreover, PSR is an invasive procedure that requires a precise surgical technique and significant postoperative care. The surgical procedure itself can trigger an inflammatory response in certain patients.

Compared with the physical reinforcement of PSR, remodelling of the physiological structure of the sclera plays a more crucial role in the development and progression of myopia. Fibroblasts, a type of cell found within connective tissues, are crucial factors in this process. Fibroblasts are responsible for producing collagen to maintain the extracellular matrix (ECM) [11–13], which provides structural support to the sclera [14]. In addition, fibroblasts produce enzymes such as matrix metalloproteinases (MMPs) that breakdown collagen. In myopic sclera, elevated MMP activity results in reduced collagen content and compromised structural integrity, facilitating scleral expansion and axial elongation. Transplanting fibroblasts cultured *in vitro* to the sclera can trigger collagen synthesis and form a new layer of collagen fibres [6]. New collagen fibres can enhance the structure of the sclera, and the anti-remodelling process of physiological structures may effectively reduce the degree of axial elongation and myopia displacement in myopic eyes. Therefore, cell therapy provides a new approach for preventing the development of myopia. Recent studies have also demonstrated that transplanting free fibroblasts into the sclera can effectively reduce axial elongation in myopic guinea pigs [6]. However, the transplantation of free cells faces an enormous obstacle, anoikis, which may directly lead to cell therapy failure [15]. In addition, free cells also require strict storage and transportation environments.

Compared with free cells, hydrogel-encapsulated cells can maintain high vitality and normal function [16–18]. Soft injectable hydrogels, which have 3D network structures similar to those of the natural ECM for the transport of nutrients and oxygen, are good carriers for cells [19, 20]. Recent advances have led to the development of injectable hydrogels combined with cell delivery for ophthalmic diseases [21–23]. For example, injectable alginate-RGD hydrogel-encapsulated induced pluripotent stem cells (iPSCs)/embryonic stem cells (ESCs) can upregulate the expression of retinal pigment epithelium (RPE) markers and retinal ganglion cell markers [24]. These effects are expected to improve the development of retinal tissue. Garcia developed an acellular hyaluronic acid (HA)-based injectable hydrogel to control axial elongation and myopia development [25]. Therefore, fibroblast-loaded injectable hydrogels may be a promising new approach to prevent the development of myopia. In particular, when used to prevent the development of myopia, injectable hydrogels can not only deliver fibroblasts to repair the physiological structure of the sclera but also physically shape the sclera through compression to shorten the eye axis. This physical/physiological synergistic effect of anti-scleral remodelling may lead to better therapeutic outcomes.

**Figure 1.**
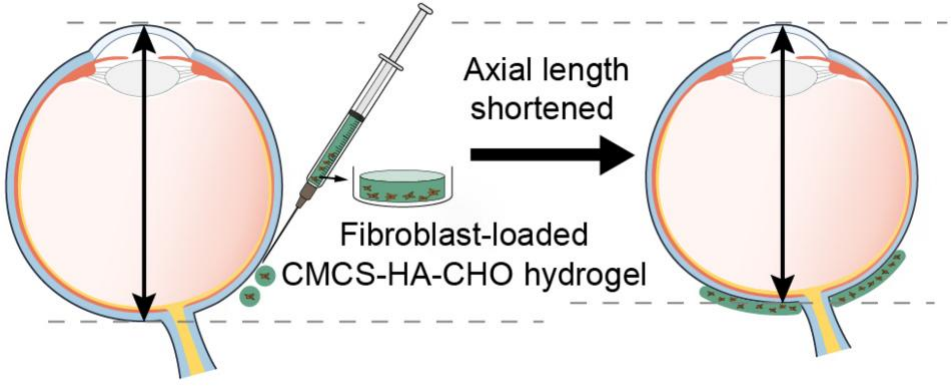
The mechanism by which injectable fibroblast-loaded CMCS-HA-CHO hydrogel promotes anti-scleral remodelling to prevent the development of myopia.

In our research, by combining hydrogels and fibroblasts, we aimed to develop a creative material consisting of an injectable hydrogel composed of carboxymethyl chitosan (CMCS) and aldehyde HA (HA-CHO) loaded with fibroblasts to prevent the development of myopia (Figure 1). By focusing on high biocompatibility and cell delivery, the development of advanced materials for PSR can significantly improve surgical outcomes and therapeutic efficacy. On the basis of the scleral remodelling mechanisms of fibroblasts, the transplantation of fibroblast-loaded hydrogels offers a promising avenue for therapeutic intervention to prevent or slow the progression of myopia. The hydrogel can conform to the scleral surface and provide a supportive matrix for cell growth, which will enhance the strength of the scleral collagen. Additionally, injectable hydrogels can be administered through a syringe, which requires only a small incision or needle puncture. This minimally invasive approach reduces medical surgical malpractice, shortens recovery time, and reduces the risk of complications associated with larger incisions.

## Materials and methods

### Materials

CMCS (chitosan derivatives, disaccharide units: N-acetylglucosamine and glucosamine, deacetylation degree >90%, O-carboxymethyl, carboxymethylation degree 80%, viscosity: 10–80 mPa·s, molecular weight: 150–200 kDa) was purchased from MeilunBio (Dalian, China). HA (disaccharide units: D-glucuronic acid and N-acetylglucosamine, 800–1000 kDa, 98%) and ethylene glycol (98%) were purchased from Heowns (Tianjin, China). Sodium periodate (98%) was purchased from Aladdin (Shanghai, China). Dulbecco’s Modified Eagle Media (DMEM), fetal bovine serum (FBS), penicillin/streptomycin (P/S) were purchased from Gibco (Shanghai, China). All other analytical reagents were used as received.

### Culture of fibroblasts

Mouse fibroblasts (L929) were purchased from BFB Company (Shanghai, China). Fibroblasts were cultured in complete medium (DMEM with 10% FBS and 1% P/S) on tissue culture plate (TCP) surface. And fibroblasts were cultured in the cell incubator at 37°C with high humidity and 5% CO_2_. The cells were digested with 0.25% trypsin for passaging or using when they reached 80% confluency.

### Synthesis of HA-CHO

HA-CHO was synthesized according to the reported literature [26]. Firstly, HA (1.5 g) was dissolved in deionized water (200 mL) at room temperature. Then, sodium periodate solution (0.25 M, 16.5 mL) was added to the HA solution, and the reaction proceeded for 3 h in the dark. Next, ethylene glycol (1.5 mL) was added to the mixture and stirred for another 1 h to terminate the reaction. Finally, the mixture was dialyzed (8 kDa cutoff dialysis membranes) in deionized water for 3 days, and HA-CHO was obtained by lyophilization. The successful synthesis of HA-CHO was characterized by ^1^H-NMR spectra on a nuclear magnetic resonance (NMR) spectrometer (500 MHz, Bruker).

Determination of HA-CHO oxidation degree [27]: firstly, 8.69 g hydroxylamine hydrochloride was dissolved in 75 mL deionized water. And 3 mL 0.05 wt% methyl orange reagent was added to the above solution. Then the mixed solution (solution I) was diluted to 500 mL with deionized water. Respectively, 50 mg of HA and HA-CHO was dissolved in 25 mL solution I and then stirred at room temperature for 4 h. Finally, titrate the released hydrochloric acid with 0.1 mol/L sodium hydroxide solution until the endpoint of red to yellow is reached. The related reactions and calculation formula of the HA-CHO oxidation degree is as follows:


(1) $$\text{HA}-\left(\text{CHO}\right)n+{n\;H}_{2}N-\text{OH}•\text{HCl}\to \text{HA}-\left(\text{CH}=N-\text{OH}\right)n+{\text{nH}}_{2}O+\text{nHCl}$$

(2) $$\text{HCl}+\text{NaOH}\to \text{NaCl}+H_{2}O$$

(3) $$\text{Oxidation}\;\text{degree}=379\Delta V\times c\times 10^{-3}/2w,$$

where ΔV (mL) is the volume difference between the sodium hydroxide solution consumed by HA-CHO and HA; c (mol/L) is the concentration of sodium hydroxide solution; w (g) is the weight of HA-CHO; 379 is the molecular weight of HA repeating units.

### Preparation of CMCS-HA-CHO hydrogel

Solutions of 5 wt% CMCS and 4 wt% HA-CHO were prepared in DMEM, respectively. Next, 150 μL each of the two solutions were mixed and vortexed for 30 s to form a uniform CMCS-HA-CHO hydrogel. The Attenuated Total Reflection Fourier Transform Infrared Spectroscopy (ATR-FTIR) spectra of freeze-dried HA, HA-CHO, CMCS, CMCS/HA and CMCS-HA-CHO were determined. The X-ray photoelectron spectroscopy (XPS) spectra of CMCS/HA and CMCS-HA-CHO were also determined.

In order to prepare fibroblast-loaded CMCS-HA-CHO hydrogel, fibroblasts obtained by digestion and centrifugation were resuspended in HA solution (5 × 10^6^ cells/mL). Then, 5 wt% CMCS and 4 wt% fibroblasts-loaded HA-CHO are mixed evenly to obtain an injectable fibroblasts-loaded CMCS-HA-CHO hydrogel.

### Rheological properties

The rheological properties of the hydrogels were tested on a rheometer (MCR302, Anton Paar) with a temperature-controlled Peltier plate system at 37°C. The 25-mm flat rotor was selected, and the working gap distance was set at 0.3 mm. An appropriate amount of deionized water was placed around the rheometer platform to prevent water evaporation. To monitor the dynamic modulus change of CMCS-HA-CHO hydrogel, 150 μL CMCS (5 wt%) and 150 μL HA-CHO (4 wt%) were mixed and transferred to the rheometer platform, and then the storage modulus (G′) and loss modulus (G″) of hydrogel were recorded with time. To demonstrate the stability of hydrogels, the frequency sweep test was conducted from 0.1 to 10 Hz with 1% strain, and the G′ and G″ of hydrogels were monitored. To investigate the injectability of hydrogels, the shear strain sweep was executed from 1% to 1000% with 1 Hz frequency, and the shear rate sweep was conducted from 1/s to 1000/s with 1 Hz frequency and 1% strain. Furthermore, the alternate step strain sweep (1–500–1–1000–1%) was also measured at a constant frequency (1 Hz). As a control, we also tested the rheological properties of the mixture of 5% CMCS and 4% HA (CMCS/HA).

### Scanning electron microscopy

The microstructure of the hydrogel was characterized by scanning electron microscopy (SEM) (Hitachi SU1510, Japan). The CMCS-HA-CHO hydrogel and fibroblasts-loaded CMCS-HA-CHO hydrogel were quenched in liquid nitrogen and dehydrated in the lyophilizer. The cross-section of the lyophilized hydrogels was sprayed with gold for 40 s, and the internal microscopic morphology was observed by SEM.

### Degradation of hydrogel *in vitro*

The CMCS-HA-CHO hydrogel (200 μL) was immersed in phosphate-buffered saline (PBS) solution at 37°C to evaluate its *in vitro* degradation behaviour. At designated time intervals, the hydrogel samples were collected, freeze-dried and then weighed to determine their residual mass. The percentage of residual mass was determined using the following equation:


(4) $$\text{Residual}\;\text{mass}\;\left(\%\right)=\left(W_{t}/W_{0}\right)\times 100\%,$$

where W_0_ represents the initial dry weight of the hydrogel and W_t_ denotes the dry weight at different time point.

### Biocompatibility of hydrogel *in vitro*

#### Cytotoxicity

The cytotoxicity of CMCS-HA-CHO hydrogel was determined by CCK-8 analysis. First, fibroblast suspension (200 μL, 5 × 10^4^ cells/mL) was added to a 96-well plate and cultured for 12 h to allow the cells to adhere to the wall. Then, CMCS-HA-CHO hydrogel (20 μL) was added to the complete medium and fibroblasts were further co-cultured, followed by live/dead (Calcein-AM/propidium iodide (PI)) staining and CCK-8 assay from day 1 to day 3. For CCK-8 assay, CCK-8 (20 μL) was added to each well and incubated for 2 h, then the absorbance value (OD 450 nm) of the control group and the hydrogel-treated group were recorded by the microplate reader (Infinite M200 PRO, Tecan). The procedure for live/dead staining is described in the section ‘Subcutaneous implantation of hydrogel *in vivo*’.

#### Hemolysis rate assay

Fresh rabbit blood was centrifuged at 1500 rpm for 10 min, and the lower layer of red blood cell (RBC) was taken. Dilute RBC with PBS and centrifuge again to remove the supernatant. Repeat three times until the supernatant is clear and transparent. Dilute the RBC obtained by centrifugation 20 times and use them as the original RBC suspension (5%). The CMCS-HA-CHO hydrogels of different masses were added to 500 μL of RBC suspension, followed by adding 500 μL of PBS, and incubated for 1 h at 37°C. Five hundred microlitres of RBC suspension and 500 μL of PBS were mixed as the negative control; 500 μL of RBC suspension and 500 μL of Triton X-100 (0.5%) as the positive control. All samples were centrifuged again at 1500 rpm for 10 min, and the absorbance value at 545 nm of their supernatant was measured by the microplate reader. The hemolysis rate is calculated from the following equation:


(5) $$\text{Hemolysis rate }(\%)=\left(A_{s}-A_{n}\right)/\left(A_{p}-A_{n}\right)\times 100\%,$$

where A_s_ is the absorbance value of the different samples, A_n_ and A_p_ are the absorbance values of the negative control and positive control, respectively.

### Subcutaneous implantation of hydrogel *in vivo*

Male C57 mice (6 weeks old, 20 g) were utilized for subcutaneous implantation experiments. The mice were anaesthetised using gas anaesthesia. Forty microlitres of CMCS-HA-CHO hydrogel was injected into the subcutaneous tissue on the back of mice with a 1-mL syringe. After 3 days, the mice were euthanized. Observe the inflammatory response at the implant site. And the surrounding tissues were excised and subjected to hematoxylin and eosin (H&E) staining.

### Live/dead assay of fibroblasts-loaded hydrogel

Fibroblast-loaded hydrogel was cultured *in vitro* in the cell incubator to observe the activity of encapsulated cells. The viability of fibroblast was measured by live/dead staining using Calcein-AM/PI kits. Firstly, the fibroblasts-loaded hydrogel was immersed in Calcein-AM solution for 15 min, and washed with DMEM solution for 10 min. The hydrogel then was immersed in PI solution for 10 min and washed with DMEM. Finally, the Calcein-AM/PI stained samples were observed using a fluorescence microscope (EVOS M5000).

### Animal experiments

#### Form deprivation and fibroblast-loaded hydrogel transplantation

The research involved male tricolour guinea pigs aged 3 weeks. All subjects were free from significant ocular diseases, and both eyes had similar axial diopters. All the animal experiments were complied with the guidelines of the Tianjin Medical Experimental Animal Care, and animal protocols were approved by the Institutional Animal Care and Use Committee of Yi Shengyuan Gene Technology (Tianjin) Co., Ltd (protocol number YSY-DWLL-2023362). All animal experimental procedures were carried out in accordance with the NIH (National Research Council) Guide for the Care and Use of Laboratory Animals.

Animals were randomly assigned to six experimental groups using a random number table method prior to intervention. To minimize selection bias, group allocation was concealed during the treatment phase. Each group included 12 animals (*n* = 12), and all procedures were conducted under identical environmental and handling conditions to ensure consistency across groups. Myopia was induced by applying a Cream ballon to cover the right eye, creating a form deprivation model (FDM) over a period of 28 days. The guinea pigs were housed in plastic cages with wire tops, maintained on a 12-h light/dark cycle, and provided with free access to water and vegetables daily for diet enrichment.

The animals underwent sub-Tenon’s capsule implantation surgery at the posterior pole area, performed with the aid of a surgical microscope. The guinea pigs were anaesthetised using gas anaesthesia. The surgical procedure was as follows: fibroblast-loaded hydrogel injectable hydrogel was administered to the posterior pole using a 1 mL syringe (25 G) through the inferiortemporal quadrants. Fibroblast-containing hydrogel was all freshly prepared at the time of surgery to ensure material freshness. The injection volume was the same for each one, maintained at 40 µL. During the surgery, no suture was required. Postoperatively, eye antibiotic treatment was necessary, with gatifloxacin eye drops administered once daily. According to postoperative observations, no guinea pigs died following the surgery, and the operation success rate was 100%.

To clarify group allocation and distinguish between the effects of mechanical support and cell-based therapy, the experimental design included six groups. Group 1: Normal (Normal control): no intervention; Group 2: Myopia (Myopia model): form-deprivation only; Group 3: M-Fibroblast: injection of fibroblast suspension into myopia guinea pig; Group 4: M-Hydrogel: injection of hydrogel only without cells; Group 5: M-Fibroblast-hydrogel: injection of fibroblast-loaded hydrogel; Group 6: M-Hydrogel + post-op fibroblast: hydrogel + post-op fibroblast injection. Each treatment group was given a single injection administration.

#### Eye axis length measurements

Each group underwent a series of eye axis length measurements at one of the five time points (0, 1, 2, 3, 4 weeks) using MD-1000A Ulthrasonic Biometer. To make these measurements, all guinea pigs were anaesthetised under general anaesthesia with gas anaesthesia, and their pupils were dilated with three drops of 0.5% phenylephrine. To ensure measurement precision and reliability, ocular biometric parameters (axial length and equatorial diameter) were measured using A-scan ultrasonography and standardized digital calliper-assisted imaging. Each eye was measured three times, and the average value was recorded. To assess inter-observer reliability, two trained examiners independently performed measurements in a masked manner on randomly selected samples (20% of total eyes), and the inter-class correlation coefficient (ICC) was calculated, yielding values >0.95, indicating excellent agreement. The ultrasound device was calibrated weekly using a manufacturer-supplied phantom to ensure consistency, and intra-instrumental error was maintained within ±10 μm.

#### Intraocular pressure measurements

After injection of the fibroblast-loaded hydrogel, the intraocular pressure (IOP) of guinea pigs was measured using a rebound tonometer (FA-800vet) throughout the 4-week experimental period.

#### SEM analysis and biomechanics measurements of sclera

The scleras of the sacrificed guinea pigs were fixed with electron microscopy fixative (2.5% glutaraldehyde) and freeze-dried. The samples were observed under SEM (Hitachi SU1510) after gold spraying. And the biomechanics of the sclera were measured using an electronic universal testing machine (Guanten, WDW-50N). In brief, the sclera was cut into rectangular strips with a width of approximately 1 mm and subjected to tensile testing at a rate of 50 mm/min. The tensile modulus (0–5% strain) and tensile strength are calculated based on the tensile stress-strain curve.

#### Histological assessment of the sclera

Guinea pigs were euthanized at week 4, and their eyeballs were fixed in 4% paraformaldehyde. The eyeballs were embedded in paraffin and sliced, and the sections (5 μm) were stained with H&E for histological analysis. For immunofluorescence staining, paraffin sections were deparaffinized and rehydrated. Then the sections were incubated with the primary antibodies against collagen type I (Col I, 1:150, Abcam) and secondary antibody goat anti-mouse (1:200, Abcam), followed by the co-staining with DAPI. The H&E images and Col I/DAPI immunofluorescence images all scanned by a panoramic scanner (3DHISTECH P250 FLASH).

### Statistical analyses

All statistical analyses were performed using SPSS 27.0. One-way analysis of variance (ANOVA) or repeated-measures ANOVA was used for comparisons among multiple groups, depending on the nature of the data. When appropriate, LSD post hoc tests were applied under the assumption of homogeneity of variances. For longitudinal variables measured at multiple time points (e.g. axial length and equatorial diameter), repeated-measures ANOVA were employed to account for within-subject correlations.

All data were presented as mean ± standard deviation (SD), with *P* < 0.05 considered statistically significant. In addition to *P*-values, effect sizes (Cohen’s d) and 95% confidence intervals (CIs) were reported to improve the interpretation of both statistical and clinical relevance. In this study, the initial number of animals was set to 12, including the sample that needed to euthanize some guinea pigs at 2 weeks to calculate the visual axial length of their eyeballs. This initial number ensures that the A-scan monitoring data at each time point is ≥6 samples for effective statistical analysis.

## Results and discussion

### Preparation and characterization of CMCS-HA-CHO hydrogel

Both CMCS and HA are animal-derived polysaccharide polymers with good biocompatibility. CMCS is a water-soluble derivative of chitosan that promotes fibroblast proliferation and accelerates wound healing [28, 29]. HA is an ECM component with a unique molecular structure and physiological functions, such as lubricating joints, regulating vascular wall permeability, and promoting cell proliferation and wound healing [30, 31]. CMCS and HA are the most common biomaterials used to treat ophthalmic diseases, and they have been widely used for corneal injury repair and as vitreous substitutes [32, 33]. Therefore, CMCS and HA have good biocompatibility and biosafety as ophthalmic implant materials.

**Figure 2.**
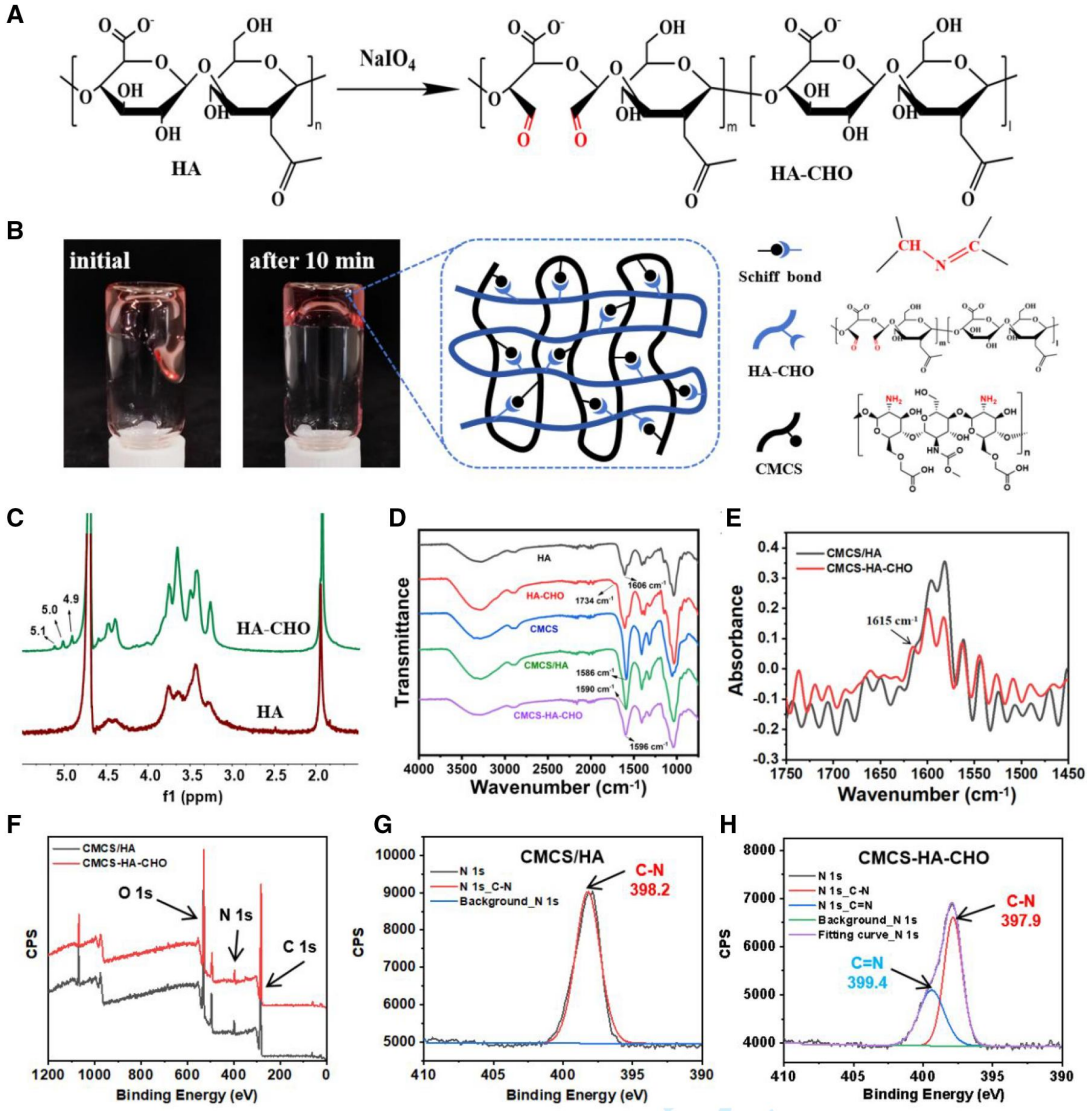
Preparation and characterization of CMCS-HA-CHO hydrogel. (**A**) The synthetic pathway of HA-CHO. (**B**) Chemical structure and formation of CMCS-HA-CHO. (**C**) ^1^H-NMR spectra of HA and OHA in D_2_O. (**D**) ATR-FTIR spectra of HA, HA-CHO, CMCS, CMCS/HA and CMCS-HA-CHO. (**E**) Fourier self-deconvolution results of ATR-FTIR spectra of CMCS/HA and CMCS-HA-CHO. (**F**) XPS wide scan spectra of CMCS/HA and CMS-HA-CHO. (**G**) High-resolution N 1 s spectrum of CMCS/HA. (**H**) High-resolution N 1 s spectrum of CMCS-HA-CHO.

Aldehyde HA (HA-CHO) is prepared by the oxidation of HA with sodium periodate. The adjacent hydroxyl groups of HA undergo ring opening to form two aldehyde groups under oxidation (Figure 2A). The CMCS molecular chain is rich in amino functional groups; thus, CMCS and HA-CHO can form a hydrogel through simple mixing on the basis of Schiff base reactions of amino and aldehyde groups (Figure 2B). The formation of CMCS-HA-CHO hydrogel is a dynamic process. As shown in Figure 2B, CMCS-HA-CHO initially had a certain fluidity after mixing, and it formed a solid hydrogel as the reaction degree increased with time. The successful synthesis of HA-CHO and the formation of CMCS-HA-CHO hydrogel were confirmed by ^1^H-NMR and ATR-FTIR analysis. As shown in Figure 2C, compared with those of HA, the new peaks of HA-CHO at 4.9–5.1 ppm were attributed to hydrated aldehyde groups [34]. In addition, the ATR-FTIR spectrum of HA-CHO showed a weak aldehyde group C = O peak at 1734 cm^−1^, also indicating the successful oxidation of HA-CHO (Figure 2D). The amide bond clearly presented an FTIR peak at 1606 cm^−1^ in HA and HA-CHO. Furthermore, the degree of oxidation of HA-CHO was determined to be 15.3 ± 0.9% by the hydroxylamine hydrochloride titration method, which means that HA-CHO has a low aldehyde content [27]. The CMCS and CMCS/HA samples presented peaks corresponding to amide bonds at 1586 and 1590 cm^−1^, respectively. Notably, the Schiff base bond (C=N) formed in CMCS-HA-CHO resulted in a new peak appearing near 1615 cm^−1^ compared with that of CMCS/HA, according to the Fourier self-deconvolution results (Figure 2E). However, owing to the relatively low content of the Schiff base formed, this peak was covered by the amide band, forming a broad peak at 1596 cm^−1^, which is consistent with previous research results [35]. In contrast to the amide bond peak of CMCS/HA at 1590 cm^−1^, the new broad peak of CMCS-HA-CHO was blueshifted to 1596 cm^−1^ because of the superposition effect of the imine peak (1615 cm^−1^). In addition, the spectrum of CMCS-HA-CHO did not show a peak at 1734 cm^−1^ corresponding to HA-CHO, indicating that the aldehyde group was consumed during the formation of the Schiff base. To further demonstrate the generation of Schiff base bonds in CMCS-HA-CHO, we conducted XPS analysis on CMCS/HA and CMCS-HA-CHO (Figure 2F). The high-resolution N 1-s spectra showed that compared with the C-N single peak at 398.2 eV in the CMCS/HA spectrum, a new C=N peak attributed to the Schiff base bond appears at 399.4 eV in the CMCS-HA-CHO spectrum (Figure 2G and H).

**Figure 3.**
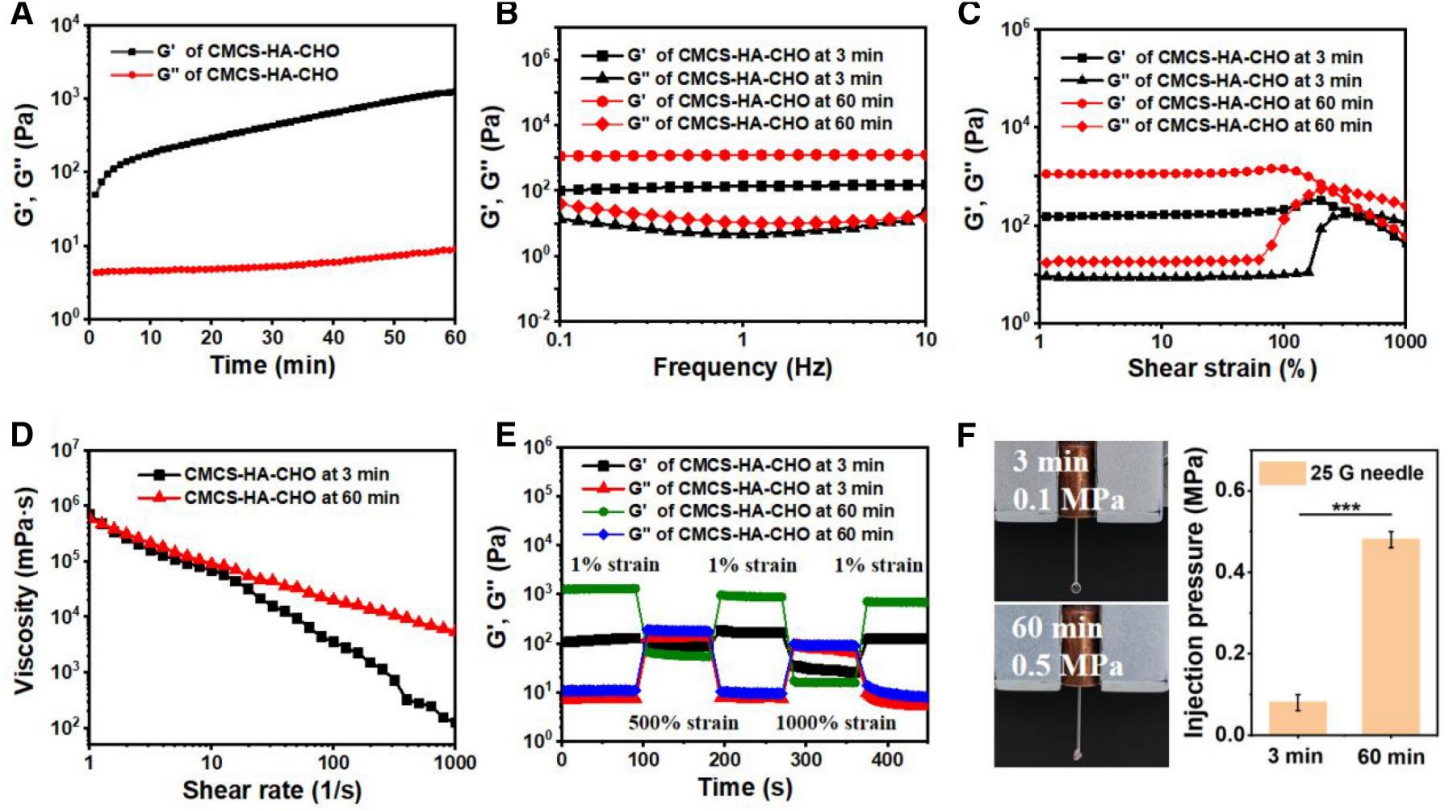
Rheological properties and injectability of CMCS-HA-CHO hydrogel. (**A**) G′ and G″ of CMCS-HA-CHO hydrogel as a function of time. (**B**) G′ and G″ of CMCS-HA-CHO hydrogel measured at 3 and 60 min under a frequency ranging from 0.1 to 10 Hz. (**C**) G′ and G″ of the CMCS-HA-CHO hydrogel measured at 3 and 60 min under a shear strain ranging from 1% to 1000%. (**D**) Viscosities of CMCS-HA-CHO hydrogel measured at 3 and 60 min under a shear rate ranging from 1/s to 1000/s. (**E**) The cyclic strain sweep of CMCS-HA-CHO hydrogel at 3 min and at 60 min. (**F**) Injection process of the hydrogel at 3 and at 60 min and their injection pressures (*n* = 4; ***P < 0.001).

We further characterized the dynamic change in the elastic modulus during the formation of the CMCS-HA-CHO hydrogel through rheological measurements. First, we recorded the change in the elastic modulus with time during the formation of the CMCS-HA-CHO hydrogel (Figure 3A). Owing to the rapid reaction of Schiff base chemistry and the required instrument start-up time, the hydrogel immediately presented a gel state (G′>G″) at the beginning of the measurement. However, at this time, the hydrogel was very soft, and its G′ was <100 Pa (within 3 min). With increasing time, the elastic modulus of the hydrogel gradually increased, and its G′ reached 1237.2 Pa at 60 min. The hydrogel always maintained a gel state (G′>G″) in the frequency range of 0.1–10 Hz at 3 and 60 min (Figure 3B), which indicated that it could maintain a stable gel state at the blink frequency when injected into the eye. The average G′ value of the hydrogel at 60 min was 1205.3 Pa, which was significantly greater than that of the hydrogel at 3 min (131.2 Pa).

Owing to its low modulus, the initial CMCS-HA-CHO hydrogel could be easily extruded through a 1 mL syringe (26 G), as shown in Supplementary Figure S1. We explored the injectable behaviour of the hydrogels through rheological tests. The strain sweep curves revealed that at 3 and 60 min, the CMCS-HA-CHO hydrogels all exhibited a gel state (G′>G″) in the low strain range (1–100%) and a sol state (G′<G″) at a high strain of 1000% (Figure 3C). This finding indicates that the dynamic Schiff base network of the CMCS-HA-CHO hydrogel will dissociate under high strain to achieve injectability. Although the hydrogels both showed injectable behaviour at 3 and 60 min, the G″ of the hydrogels at 60 min was significantly greater than that at 3 min under high strain. The strain rate sweep of the hydrogels showed a similar trend. As shown in Figure 3D, at both 3 and 60 min, the hydrogels showed shear thinning behaviour, but the viscosity of the hydrogels at 60 min was also significantly greater than that at the high shear rate. These results indicate that the initial CMCS-HA-CHO hydrogel had better injectable behaviour. In addition, alternate step strain sweep measurements were performed to simulate the injection process of the CMCS-HA-CHO hydrogel. As shown in Figure 3E, CMCS-HA-CHO underwent a gel–sol transition when the shear strain changed from 1% to 500%. When the shear strain recovered to 1%, CMCS-HA-CHO immediately returned to the gel state (G′ > G″), indicating rapid reconstruction of the Schiff base bond. Even when the strain increased to 1000%, the hydrogel could also achieve a reversible gel–sol–gel transition, which means that the hydrogel was thixotropic under large strain changes. This thixotropy endows the hydrogel with good injectability. Notably, at high shear strains (500% and 1000%), the loss modulus G″ of the hydrogel at 60 min was significantly greater than that of the hydrogel at 3 min. A larger loss modulus requires the material to consume more energy during deformation, which means that greater resistance will be generated during the injection process [36]. Furthermore, we directly measured the injection pressure of the CMCS-HA-CHO hydrogel at 3 and 60 min using a pneumatic device within a 3D printer. As shown in Figure 3F, the average injection pressure of the CMCS-HA-CHO hydrogel at 3 min was very low (0.08 MPa), and the extruded hydrogel resembled a water drop; however, at 60 min, the CMCS-HA-CHO hydrogel presented a high average injection pressure (0.48 MPa), and the extruded hydrogel was broken.

As a control, we also studied the rheological properties of a mixture (CMCS/HA) composed of CMCS and unmodified HA. As shown in Supplementary Figure S2A, due to the high viscosity of high-molecular-weight HA, CMCS/HA exhibited an unstable gel state (G′ > G″) under low strain (0.1%). As the strain increased, G′ gradually exceeded G″ (>1%), and CMCS/HA presented a sol state (G′ < G″). At a low oscillation frequency (<1 Hz), CMCS/HA exhibited a viscous sol state; at a high oscillation frequency (10 Hz), CMCS/HA presented an elastic gel state (Supplementary Figure S2B). As shown in Supplementary Figure S2C, at 1% strain and 1 Hz, CMCS/HA consistently exhibited a sol state (G′ < G″). The inverted experiment also confirmed that CMCS/HA was in a flowable viscous state. These results indicate that CMCS/HA cannot form stable hydrogels with physical interactions and that the chemical crosslinking formed by the Schiff base in the CMCS-HA-CHO hydrogel is necessary.

### Biocompatibility of CMCS-HA-CHO

**Figure 4.**
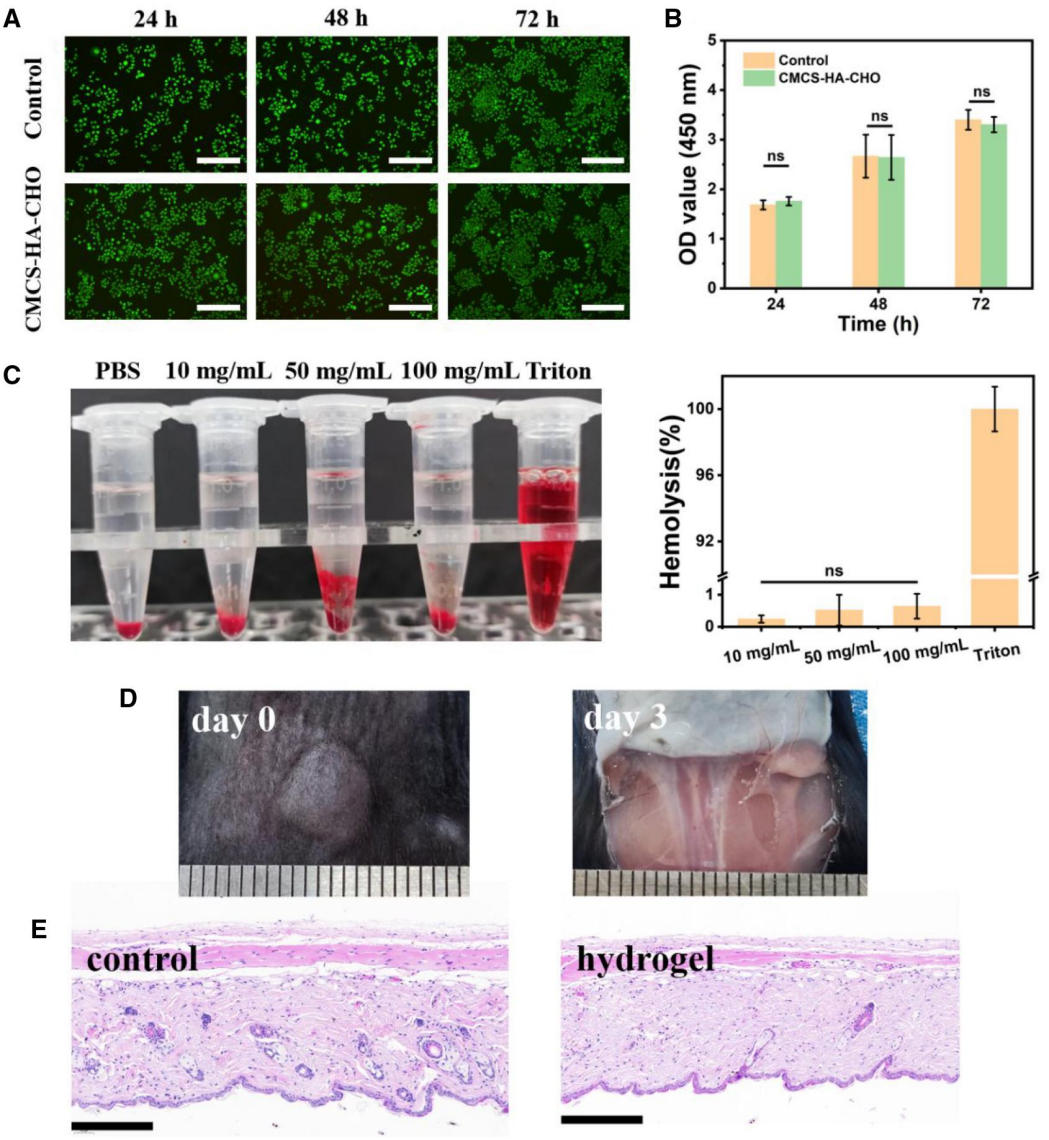
Biocompatibility of CMCS-HA-CHO. (**A**) Live/dead staining images of fibroblasts treated with CMCS-HA-CHO hydrogels and the control group at 24, 48, 72 h. Bar: 300 μm. (**B**) CCK-8 assays of rASCs fibroblasts treated with CMCS-HA-CHO hydrogels and the control group at 24, 48, 72 h (*n* = 6; ns: no significance). (**C**) Haemolyses rate of CMCS-HA-CHO hydrogels of different concentrations (*n* = 4; ns: no significance). (**D**) Subcutaneous implantation of CMCS-HA-CHO hydrogel in C57 mice. Bar: 1 mm. (**E**) H&E images of the normal skin tissue and the skin tissue implanted with hydrogel. Bar: 200 μm.

First, the cytocompatibility of the CMCS-HA-CHO hydrogel *in vitro* was evaluated by live/dead staining and CCK-8 analysis. As shown in Figure 4A, the fibroblasts in both the control group and the hydrogel-treated group maintained good activity after 72 h of culture, and the cell density increased continuously. The fibroblasts had good cellular growth behaviour and tended to exhibit a spindle-shaped cell pattern. In addition, there was no significant difference in cell growth status between the control group and the hydrogel-treated group at 24, 48 or 72 h, which fully confirmed the good cytocompatibility, with a cell survival rate >80% (Figure 4B) [37]. Additionally, the haemolysis of the CMCS-HA-CHO hydrogel was measured through RBC incubation, as shown in Figure 4C. The supernatants of hydrogels with different mass concentrations (10, 50 and 100 mg/mL) incubated with RBCs were clear and transparent. They all showed negligible haemolysis rates (<1%), which meets the requirement for a clinically safe haemolysis rate (<5%) [38]. These *in vitro* results demonstrated that the CMCS-HA-CHO hydrogel could be used as a type of biosafety ophthalmic implant with good cytocompatibility and a low haemolysis rate.

Furthermore, we verified the biocompatibility of the CMCS-HA-CHO hydrogel *in vivo* through subcutaneous implantation. As shown in Figure 4D, the hydrogel had basically degraded after 3 days of implantation, and there was no sign of an inflammatory reaction in the subcutaneous tissue of the mice. We characterized the degradation behaviour of the hydrogel *in vitro*. As shown in Supplementary Figure S3, the hydrogel exhibited rapid degradation kinetics in PBS at 37°C. This material demonstrated a mass retention of 64.7 ± 3.6% at 12 h, followed by continuous degradation to 21.2 ± 2.8% residual mass at 48 h, ultimately achieving complete degradation by 60 h. In addition, H&E staining was performed on skin tissue implanted with the hydrogel and normal tissue. As shown in Figure 4E, there was no obvious increase in the number of inflammatory cells in the hydrogel group compared with that in the control group (normal tissue), which also confirmed the good biocompatibility of the hydrogel *in vivo*.

### Fibroblast-loaded CMCS-HA-CHO hydrogel

**Figure 5.**
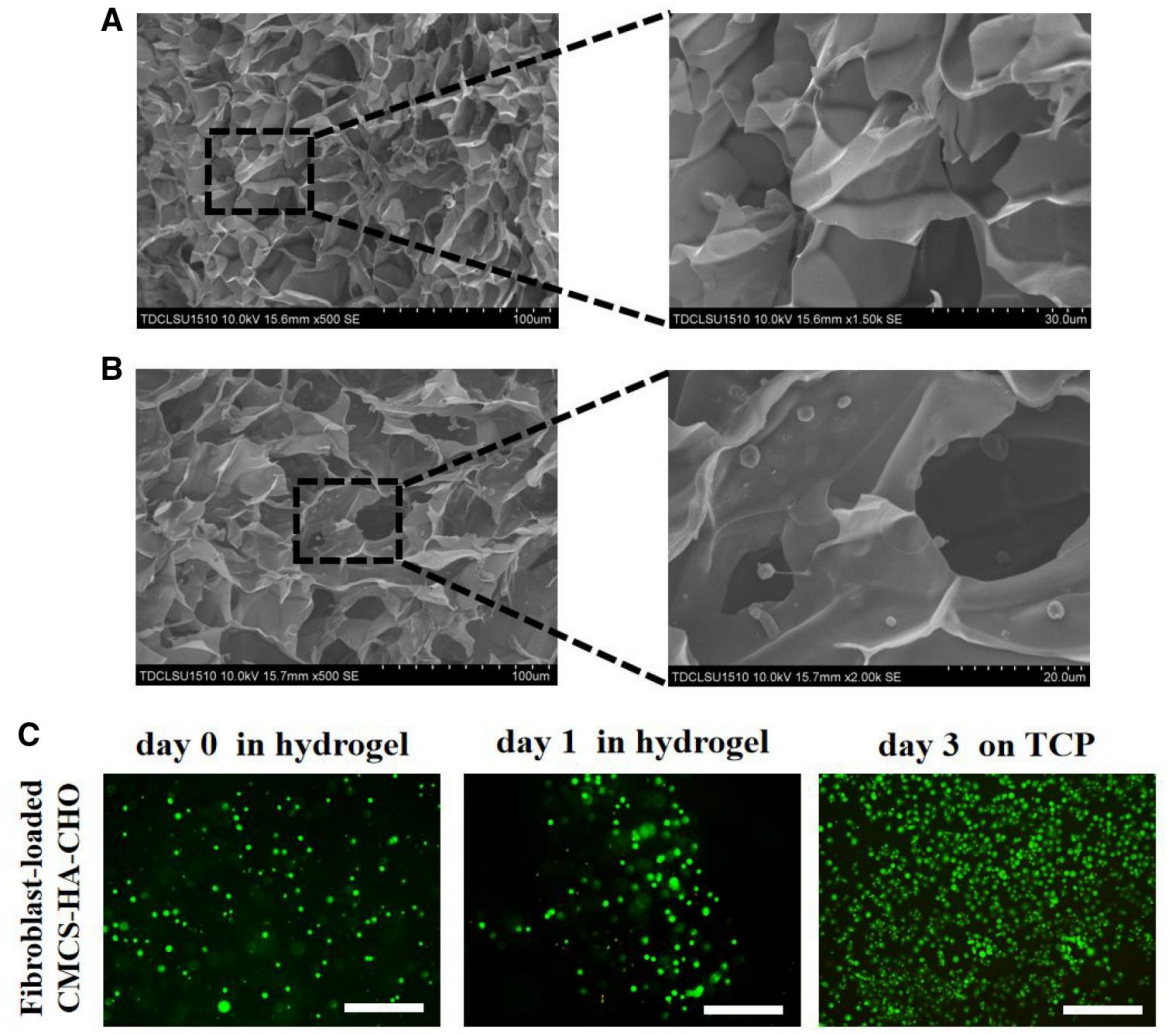
Fibroblasts-loaded CMCS-HA-CHO hydrogel. SEM morphologies of (**A**) CMCS-HA-CHO hydrogel and (**B**) fibroblasts-loaded CMCS-HA-CHO hydrogel at different magnifications. (**C**) Live/dead staining of fibroblasts in CMCS-HA-CHO hydrogel at day 0 and day 1 *in vitro*, and scattered fibroblasts adhered to TCP at day 3. Bar: 300 μm.

We prepared an injectable fibroblast-loaded CMCS-HA-CHO hydrogel for anti-scleral remodelling. In brief, the fibroblasts obtained by digestion and centrifugation were suspended in sterile HA-CHO solution and mixed with sterile CMCS solution to form a fibroblast-loaded CMCS-HA-CHO hydrogel. The survival of encapsulated fibroblasts is crucial to the biological function of hydrogels after implantation into the eye. First, we observed the microstructures of the hydrogel and the fibroblast-loaded hydrogel. As shown in Figure 5A, the CMCS-HA-CHO hydrogel had an interconnected porous network structure. In addition, the fibroblasts were randomly distributed in the fibroblast-loaded hydrogel network (Figure 5B). This network structure is conducive to the transportation of oxygen and nutrients and can provide 3D growth space for encapsulated cells, promoting cell adhesion, proliferation, and cytokine secretion.

Furthermore, we explored the cell viability in the presence of the injectable fibroblast-loaded CMCS-HA-CHO hydrogel through live/dead staining. As shown in Figure 5C, the cells in the fibroblast-loaded hydrogel extruded by a 1 mL syringe needle (26 G) still maintained high activity, and there were almost no dead cells. These findings indicate that the soft CMCS-HA-CHO hydrogel (3 min) did not produce excessive shear stress on the cells during extrusion and did not decrease cell survival. After one day of *in vitro* culture, the cells in the hydrogel still maintained good viability, with only a few dead cells. The diameter of the fibroblasts increased significantly, which means that the fibroblasts grew well in the hydrogel. On day 3, the hydrogel had degraded, and the encapsulated fibroblasts were scattered on the tissue culture plate (TCP) to adhere and grow, maintaining good cellular activity. These results indicate that hydrogel-encapsulated fibroblasts can maintain good growth status after injection and still maintain cell viability after degradation of the hydrogel.

### *In vivo* assessment of fibroblast-loaded hydrogel in the myopia model

**Figure 6.**
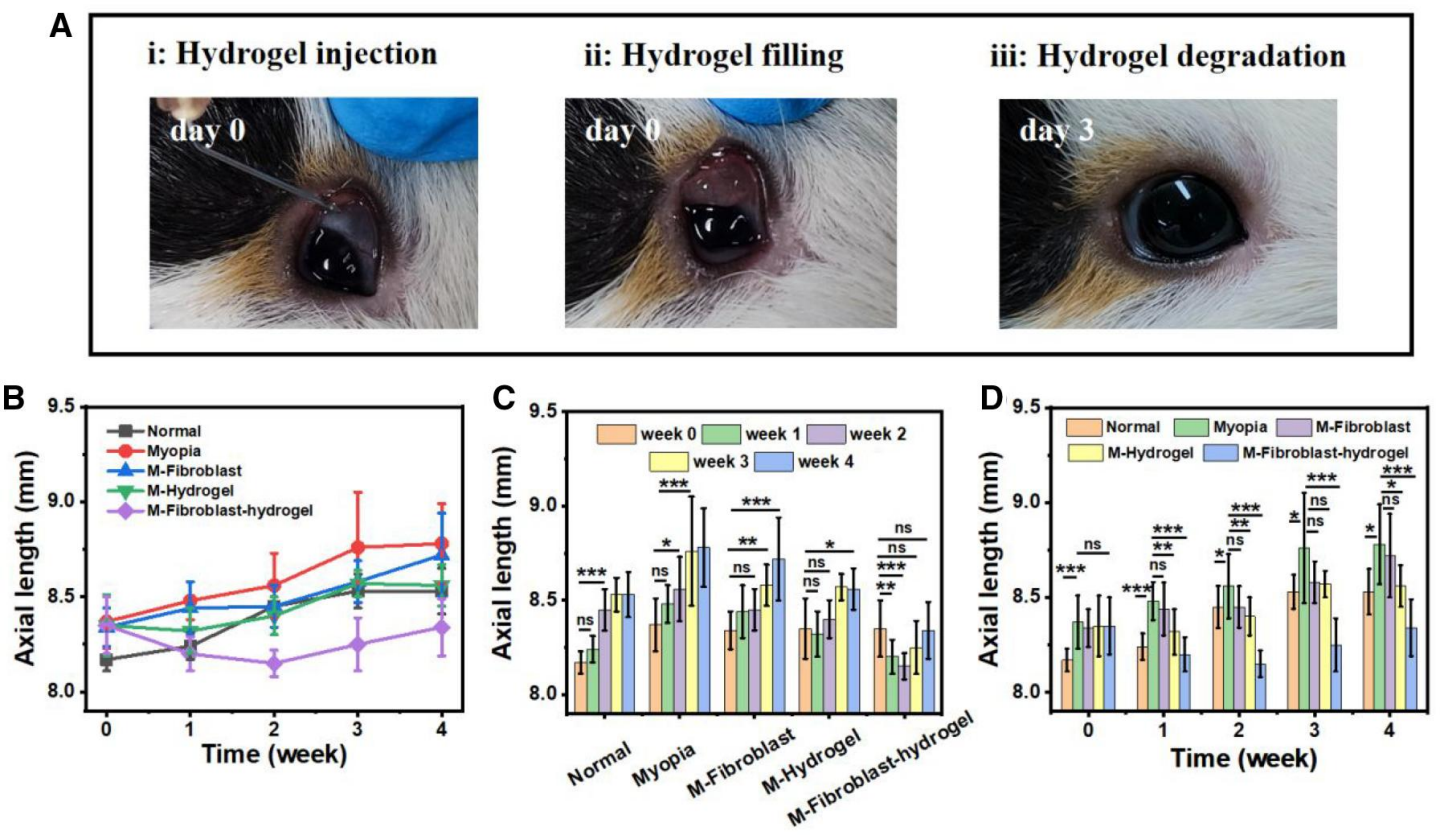
Surgical procedure of hydrogel and change of eye axial length in each group. (**A**) Implantation of injectable fibroblast-loaded CMCS-HA-CHO hydrogel into the sclera. (**B**–**D**) Change of eye axial length over 4 weeks in each group (*n* ≥ 6; ns: no significance, *P < 0.05, **P < 0.01, ***P < 0.001).

The myopia model used in the animal experiments was a FDM. After 4 weeks of monocular FDM, significant differences (*P* < 0.001) in axial length between binoculi were found, and the axial length of the covered eye (8.37 ± 0.14 mm) was significantly greater than that of the normal eye (8.17 ± 0.06 mm). Guinea pigs in which an FDM model was successfully established were divided into six groups as follows: normal growth without any intervention (Normal, N), right eye covered for 4 weeks (Myopia, M), myopia treated with fibroblasts (M-Fibroblast), myopia treated with hydrogel (M-hydrogel), myopia treated with fibroblast-loaded hydrogel (M-Fibroblast-hydrogel), and myopia treated with hydrogel + post-op fibroblast injection (M-hydrogel + post-op fibroblast). The operation process is shown in Figure 6A. Forty microlitres of fibroblast-loaded CMCS-HA-CHO hydrogel was injected into the posterior sclera of guinea pigs. Three days after transplantation, the hydrogel had fused with the sclera, and there was no abnormality in the eyeball.

We recorded the changes in the axial length of guinea pigs in each group within 4 weeks using an ultrasonic biometer. As displayed in Figure 6B–D and Supplementary Table S1, the axial length of the Normal and Myopia groups gradually increased with the growth of guinea pigs within 4 weeks. The axial length in the Myopia group was always greater than that in the Normal group, which was due to the increase in axial length caused by changes in the physiological structure of the sclera in the myopia group. The trend of axial length change in the M-Fibroblast group was basically consistent with that in the Myopia group. Although there was a difference in values, there was no significant difference at any of the four time points (weeks 1–4). The poor therapeutic effect of free fibroblast transplantation may be due to the low cell survival rate [39] because the separation of free cells from the ECM can cause apoptosis.

The axial lengths of the M-hydrogel (8.32 ± 0.12 mm) and M-Fibroblast-hydrogel (8.20 ± 0.09 mm) groups were significantly shorter than that of the Myopia (8.48 ± 0.10 mm) group at week 1. This is because the sclera is physically shaped immediately after the implantation of the hydrogel; that is, the sclera slightly shrinks inwards under compression by the hydrogel. In addition, the implanted chitosan and HA components can also promote the growth of fibroblasts to improve the physiological structure of the sclera. The axial length of the M-hydrogel group also increased with time. At week 3, no significant difference was found in the axial length between the M-hydrogel (8.57 ± 0.07 mm) and Myopia (8.76 ± 0.29 mm) groups. These results prove that the implantation of pure CMCS-HA-CHO can delay axial elongation for only a short time. Surprisingly, the axial length of the M-Fibroblast-hydrogel group (8.34 ± 0.15 mm at week 4) was always shorter than that of the Myopia group (8.78 ± 0.21 mm at week 4) during the 4-week observation period. The implantation of the fibroblast-loaded CMCS-HA-CHO hydrogel had an excellent effect on controlling the axial elongation of myopic eyes.

We included a hydrogel + post-op fibroblast injection group in the animal studies to decouple the effects of the physical support and delivery of the hydrogel. As shown in Supplementary Figure S4, the hydrogel + postoperative fibroblast injection treatment group also showed inhibition of axial elongation at week 1 (the axial length changed from 8.44 ± 0.10 mm at week 0 to 8.30 ± 0.09 mm at week 1). However, the axial length continued to increase from week 2, suggesting the limited efficacy of the hydrogel + postoperative fibroblast injection in controlling the development of myopia. Interestingly, the therapeutic effect and trend observed in the hydrogel + postoperative fibroblast injection group were very similar to those of the hydrogel-alone treatment group. These results demonstrate the necessity of encapsulating fibroblasts within the hydrogel matrix, as cells in the hydrogel + postoperative fibroblast injection group failed to exert long-term therapeutic effects, similar to those in the fibroblast injection-alone group. And the fibroblast-loaded hydrogel group showed a more long-term effect in controlling the axial elongation.

**Figure 7.**
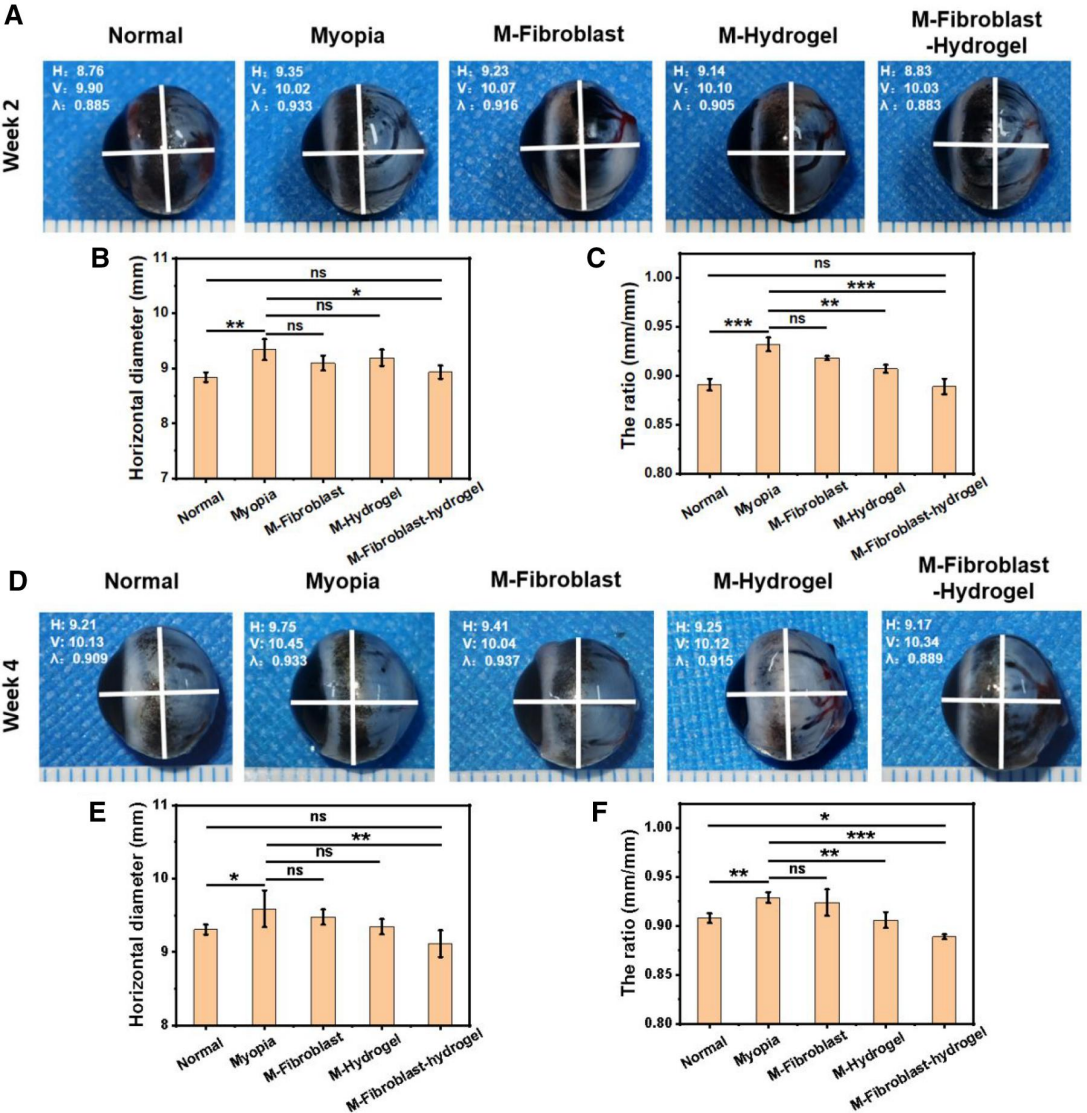
Eye images of sacrificial Guinea pigs in each group at week 2 and week 4. (**A**) Eye images, (**B**) eye axial length and (**C**) the ratio of axial length to equatorial diameter of sacrificial Guinea pigs in each group at week 2 (*n* = 3; ns: no significance, *P < 0.05, **P < 0.01, ***P < 0.001). (**D**) Eye images, (**E**) eye axial length and (**F**) the ratio of axial length to equatorial diameter of sacrificial Guinea pigs in each group at week 4 (*n* = 4; ns: no significance, *P < 0.05, **P < 0.01, ***P < 0.001).

In addition to observing axial length using an ultrasonic biometer, we measured the vertical and horizontal diameters of the eyeballs in each group after the guinea pigs were euthanized and dissection was performed at weeks 2 and 4. We used two indicators, the horizontal diameter and the ratio of the horizontal diameter to the vertical diameter, to evaluate the overall shape of the eyeball. As shown in Figure 7A–C, the horizontal diameter (9.34 ± 0.19 mm) and ratio (0.93 ± 0.01) of the eyeball in the Myopia group were significantly greater than those in the Normal group (8.84 ± 0.09 mm; 0.89 ± 0.01) at week 2 because of the increase in the axial lengths of myopic eyes. The horizontal diameters of the M-Fibroblast group (9.10 ± 0.14 mm) and M-hydrogel group (9.19 ± 0.15 mm) were not significantly decreased, but there was a significant difference in the ratios. Importantly, the horizontal diameter (8.93 ± 0.12 mm) and ratio (0.89 ± 0.01) of the M-Fibroblast-hydrogel group were significantly reduced, indicating that the M-Fibroblast-hydrogel group exhibited the best therapeutic effect. Similarly, the M-Fibroblast-hydrogel group also had the lowest horizontal length and ratio at week 4 (Figure 7D–F), again confirming the long-term therapeutic effect of the fibroblast-loaded CMCS-HA-CHO hydrogel. If only the horizontal diameter decreases but the vertical diameter increases after treatment, this indicates that surgery acts only on mechanical support and that the physiological structure of the sclera is not restored. Interestingly, the horizontal length of the eyeball in the M-Fibroblast-hydrogel group was shortened, but the vertical length did not change significantly (Figure 7A and D), suggesting that the physiological structure of the sclera had been restored.

In addition, we studied the long-term biocompatibility of the fibroblast-loaded hydrogel, including its effects on IOP and retinal integrity. As shown in Figure S5, implantation of the fibroblast-loaded hydrogel did not significantly affect the IOP. During the 8-week follow-up period, the IOP values remained within the normal physiological range (10–21 mmHg), with only minor fluctuations observed. H&E staining analysis at week 8 revealed that the retinal tissue architecture of the fibroblast-loaded hydrogel treatment group was well preserved, exhibiting clearly discernible laminar structure and an absence of obvious inflammatory infiltration, comparable to that observed in the normal control group (Supplementary Figure S6).

### Biomechanical and histological analysis of sclera

**Figure 8.**
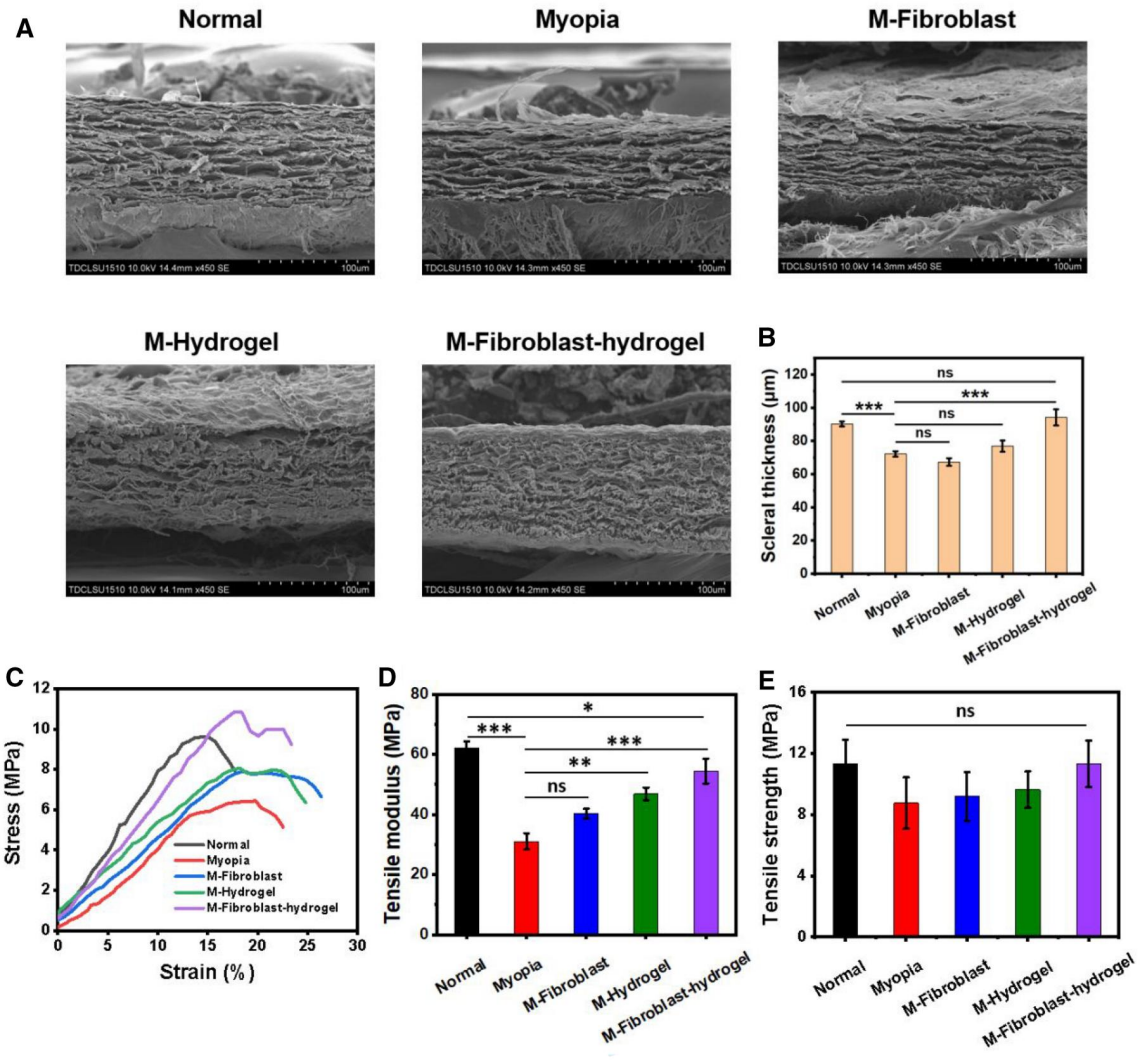
(**A**) SEM images of sclera and (**B**) scleral thickness in each group at week 4 (*n* = 4; ns: no significance, ***P < 0.001). (**C**) Tensile stress-strain curve of sclera in each group at week 4. (**D** and **E**) Tensile modulus (0–5% strain) and tensile strength of sclera in each group at week 4 (*n* = 3; ns: no significance, *P < 0.05, **P < 0.01, ***P < 0.001).

We further studied the effects of fibroblast-loaded CMCS-HA-CHO hydrogel transplantation on the tissue structure and biomechanics of the sclera at week 4. First, we observed the internal tissue structure of the sclera in each group of guinea pigs using SEM. As shown in Figure 8A and B, the sclera thickness of the Myopia group (72.25 ± 1.61 μm) was significantly lower than that of the Normal group (90.34 ± 1.49 μm) because of pathological structural changes. The scleral thicknesses of the M-Fibroblast group (67.37 ± 2.30 μm) and M-hydrogel group (76.88 ± 3.40 μm) were not significantly different from that of the Myopia group. Only the scleral thickness of the M-Fibroblast-hydrogel group (94.27 ± 4.86 μm) was significantly greater after treatment, which was equivalent to that of the Normal group. Notably, the density of collagen fibres in the sclera of the M-Fibroblast-hydrogel group was significantly greater than that in the other groups (Supplementary Figure S7), implying that the transplantation of the fibroblast-loaded CMCS-HA-CHO hydrogel reshaped the physiological structure of the sclera.

The biomechanical properties of the sclera are closely related to myopia [40]. During high myopia, the biomechanical properties of the sclera deteriorate, making it prone to deformation and expansion, leading to myopia. As displayed in Figure 8C and D, the tensile modulus of the sclera in the Myopia group was significantly lower than that of the Normal group. The tensile moduli of the sclera in the M-Fibroblast and M-hydrogel groups were greater than those in the myopia group, but there was no significant difference between the M-Fibroblast and Myopia groups. Moreover, the M-Fibroblast-hydrogel group exhibited the best therapeutic effect, and the tensile modulus of the sclera was significantly greater than that of the Myopia group and only slightly lower than that of the Normal group. However, the tensile strength of the M-Fibroblast-hydrogel group was also closest to that of the Normal group and greater than that of the other three groups (Figure 8E). However, there was no significant difference in tensile strength among the five groups. The changes in the biomechanical properties of the sclera are due to the reconstruction of the physiological structure. The implantation of fibroblast-loaded CMCS-HA-CHO hydrogels can increase the synthesis and secretion of collagen, increase the compactness of the collagen fibre network of the sclera, and increase the thickness of the sclera [41, 42]. Therefore, the antistretching ability of the sclera is improved, which can inhibit the development of high myopia.

**Figure 9.**
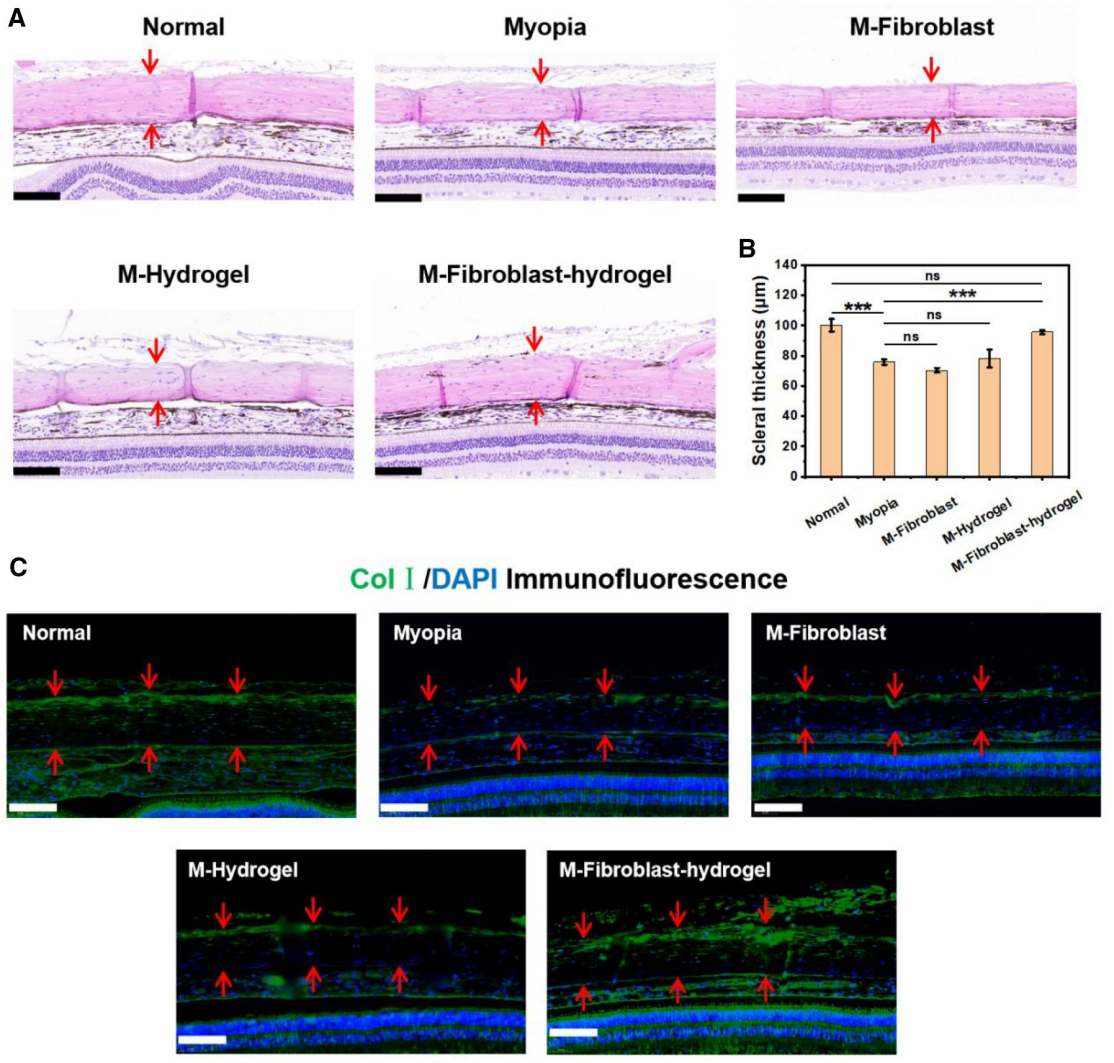
(**A**) H&E staining of the sclera (indicated by the red arrow) and (**B**) scleral thickness in each group at week 4 (*n* = 4; ns: no significance, ***P < 0.001). (**C**) Col I/DAPI immunofluorescence staining of the sclera in each group at week 4. Bar: 100 μm.

We also tested the scleral thickness in each group using H&E staining at week 4 (Figure 9A and B). Similar to the SEM results, the scleral thickness of the M-Fibroblast-hydrogel group (95.73 ± 1.38 μm) was greater than that of any of the other three groups and was similar to that of the Normal group (100.30 ± 4.07 μm). Furthermore, in Col I/DAPI immunofluorescence staining (Figure 9C and Supplementary Figure S8), the DAPI-stained fibroblast nuclei appeared bright blue, and the FITC-marked Col I was visualized in green. The overlay of the two channels revealed a high degree of colocalization, with blue nuclei surrounded by intense green collagen signals, suggesting that active fibroblasts engaged in collagen type I synthesis. Areas with weaker green signals corresponded to regions with fewer cells, indicating potential ECM degradation or less cellular activity. In the Normal group, the collagen expression in the sclera was high, whereas the collagen expression in the Myopia, M-Fibroblast and M-hydrogel groups was lower. Collagen expression in the M-Fibroblast-hydrogel group was similar to that in the Normal group and significantly greater than that in the other three groups. These results prove that CMCS-HA-CHO hydrogel-encapsulated fibroblasts can effectively participate in the synthesis of the scleral ECM and increase the thickness of the sclera, thus effectively improving the biomechanical properties of the sclera and realizing anti-scleral remodelling.

In previous studies, fibroblast transplantation and injectable hydrogel implantation have been successfully used to control axial elongation [6, 25]. The results of our study revealed that the fibroblast-loaded CMCS-HA-CHO hydrogel had a more significant and long-term effect on controlling axial elongation than CMCS-HA-CHO hydrogel implantation or free fibroblast transplantation alone. This occurred because the fibroblast-loaded CMCS-HA-CHO hydrogel repaired the physiological structure of the sclera and effectively increased its thickness. However, our study has several shortcomings. The degree of oxidation of HA-CHO is relatively low, which leads to a low degree of chemical crosslinking of CMCS-HA-CHO; therefore, the degradation rate of the CMCS-HA-CHO hydrogel *in vivo* is relatively fast. However, fibroblast-loaded CMCS-HA-CHO effectively shortened the axis during the observation period of 1 month. The long-term therapeutic effects may stem from cell therapy mediated by fibroblasts after hydrogel degradation, as single-cell therapy was also demonstrated to effectively control axial elongation in this study. Here, the CMCS-HA-CHO hydrogel provided early-stage protection postimplantation, preventing anoikis (detachment-induced apoptosis) in the transplanted cells. Moderately extending the total observation period will provide a more comprehensive and long-term evaluation of the impact of our materials on myopia. In future work, adjusting the ratio of CMCS to HA-CHO to investigate the effects of materials with different degradation rates on myopia control will be highly important. In addition, exploring alternative crosslinking strategies, such as bioorthogonal reactions, would provide valuable insights into their impact on treatment efficacy.

In the present study, we demonstrated *in vitro* that fibroblasts encapsulated within a hydrogel maintain excellent viability and that the fibroblasts released upon hydrogel degradation retain their capacity for growth and proliferation. An important consideration for future studies involves the fate of transplanted fibroblasts following hydrogel degradation. In this study, cells were still visible at the injection site on histological sections at week 4, suggesting partial survival or tissue integration. However, it remains unclear whether these cells undergo apoptosis, persist functionally, or fuse with host tissue. Determining their long-term behaviour is critical for understanding both therapeutic durability and biosafety. Future investigations incorporating lineage tracing, apoptotic markers, and immunohistochemistry (e.g. human-specific antibodies in xenografts) will be valuable for tracking cell survival, migration, and integration dynamics after scaffold degradation.

Additionally, this study used mouse fibroblasts (L929) for model research on the basis of the following scientific considerations: (1) L929 cells exhibit key fibroblast-like features, such as ECM production and responsiveness to environmental stimuli, which are functionally comparable to those of human scleral fibroblasts in many *in vitro* contexts; (2) In the form deprivation experiment, we used guinea pigs as model animals and therefore chose mouse-derived L929 cells; (3) L929 cells were chosen for this study owing to their advantages, including consistent growth and reproducibility, making them suitable for proof-of-concept studies. However, their clinical relevance remains limited. In future work, we plan to validate these findings using primary human scleral fibroblasts or fibroblast-like cells derived from iPSCs, which could offer patient-specific compatibility and reduced immunogenicity. In terms of clinical feasibility, autologous fibroblasts can be obtained from Tenon’s capsule or the conjunctival stroma using minimally invasive biopsy techniques. These cells can be expanded ex vivo for incorporation into injectable hydrogels. Further investigations, including investigations of potential inflammatory responses, T-cell activation, and long-term host–graft interactions in large animal models, are needed to assess immunologic safety.

## Conclusion

In conclusion, we prepared a fibroblast-loaded CMCS-HA-CHO hydrogel to prevent the development of myopia. The dynamic Schiff base CMCS-HA-CHO hydrogel can be injected into the posterior sclera with a low modulus, and then, the modulus increases to physically correct the ocular axis. In addition, fibroblast-loaded hydrogels can maintain a high cell survival rate and repair the scleral structure through the synergistic effect of cell therapy and mechanical support. In guinea pig models, hydrogels and fibroblast-loaded hydrogels effectively shortened the axial length of myopic eyes. The fibroblast-loaded hydrogel had a greater therapeutic effect, the scleral thickness increased significantly, and the tensile modulus increased, which proves the success of anti-scleral remodelling. This fibroblast-loaded CMCS-HA-CHO hydrogel has the advantages of minimal invasiveness and good biocompatibility and is a very promising option for preventing the development of myopia.

## Funding

This work was financially supported by the Science and Technology Foundation of Tianjin Eye Hospital (grant number YKPY2205), Tianjin Science and Technology Project (grant number 22JCZDJC00280), Tianjin Key Medical Discipline Construction (TJYXZDXK-3-004A-3).

## Supplementary Material

rbaf096_Supplementary_Data
