# Supplementary material for: Fibroblast-loaded carboxymethyl chitosan—aldehyde hyaluronic acid injectable hydrogel for scleral remodelling to prevent development of myopia
Source: Regen Biomater. 2025 Oct 10;12:rbaf096. doi: 10.1093/rb/rbaf096 (PMC12603358; doi:10.1093/rb/rbaf096)
Supplement: rbaf096_Supplementary_Data [file rbaf096_supplementary_data.docx]

**Supporting Information**

**Fibroblast-loaded carboxymethyl chitosan - aldehyde hyaluronic acid injectable hydrogel for scleral remodeling to prevent development of myopia**

Jingwen Hui ^1,2^, Kexin Tang^1,2^, Yuejun Zhou^1,2,4^, Ziming Wang^1,2^, Qian Zhang^3^, Guoge Han^1,2^, Wenguang Liu^3^, Xiongfeng Nie ^3*^, Quanhong Han ^1,2*^, Xiaoyong Yuan^1,2*^

^1^Clinical College of Ophthalmology, Tianjin Medical University, Tianjin, China

^2^Tianjin Eye Hospital, Tianjin Key Laboratory of Ophthalmology and Visual Science, Tianjin Eye Institute, Tianjin, China

^3^School of Material Science and Engineering, Tianjin Key Laboratory of Composite and Functional Materials, Tianjin University, Tianjin 300350, China.

^4^School of Medicine, Nankai University, Tianjin, China.

^*^ Corresponding author E-mail:

[yuanxy_cn@hotmail.com,](mailto:yuanxy_cn@hotmail.com,) [hanquanhong126@126.com](mailto:hanquanhong126@126.com), xfnie@tju.edu.cn


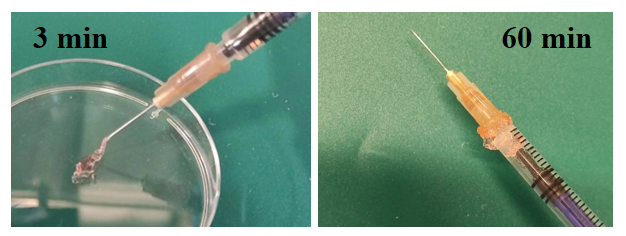


**Figure S1.** Digital image of the injection process of CMCS-HA-CHO hydrogel at 3 min and 60 min.


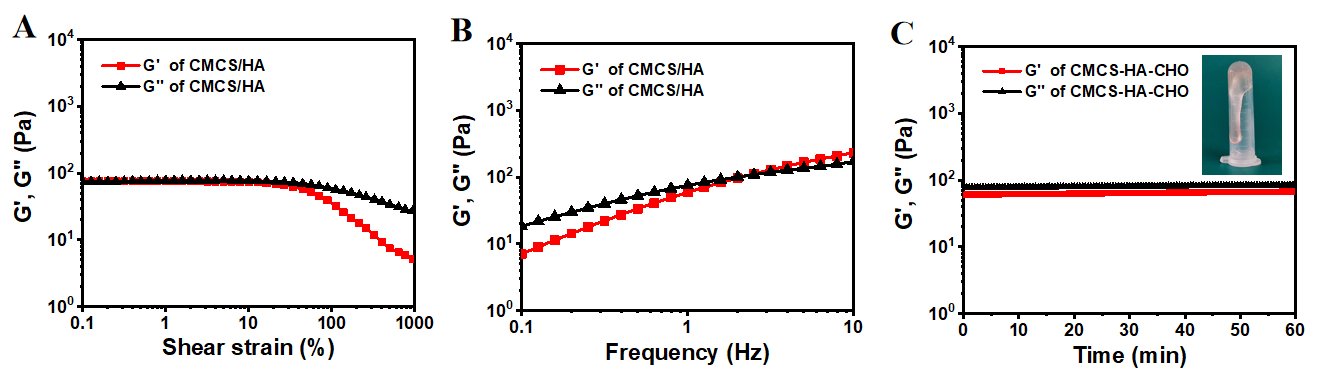


**Figure S2.** Rheological properties of CMCS/HA. (A) G' and G'' of CMCS/HA under a shear strain ranging from 0.1% to 1000% at the frequency of 1 Hz. (B) G' and G'' of CMCS/HA under a frequency ranging from 0.1 Hz to 10 Hz at the strain of 1%. (C) G' and G'' of CMCS/HA as a function of time at the strain of 1% and the frequency of 1 Hz.

**Figure S3.** The degradation curve of the CMCS-HA-CHO hydrogel in PBS at 37 °C.

**Figure S4.** Change of eye axial length over 4 weeks in the M-hydrogel + post-op fibroblast group (n ≥ 5; ns: no significance, *: p <0.05).

**Figure S5.** The intraocular pressure (IOP) after the injection of fibroblast-loaded hydrogel (n ≥ 4; ns: no significance, *: p <0.05).


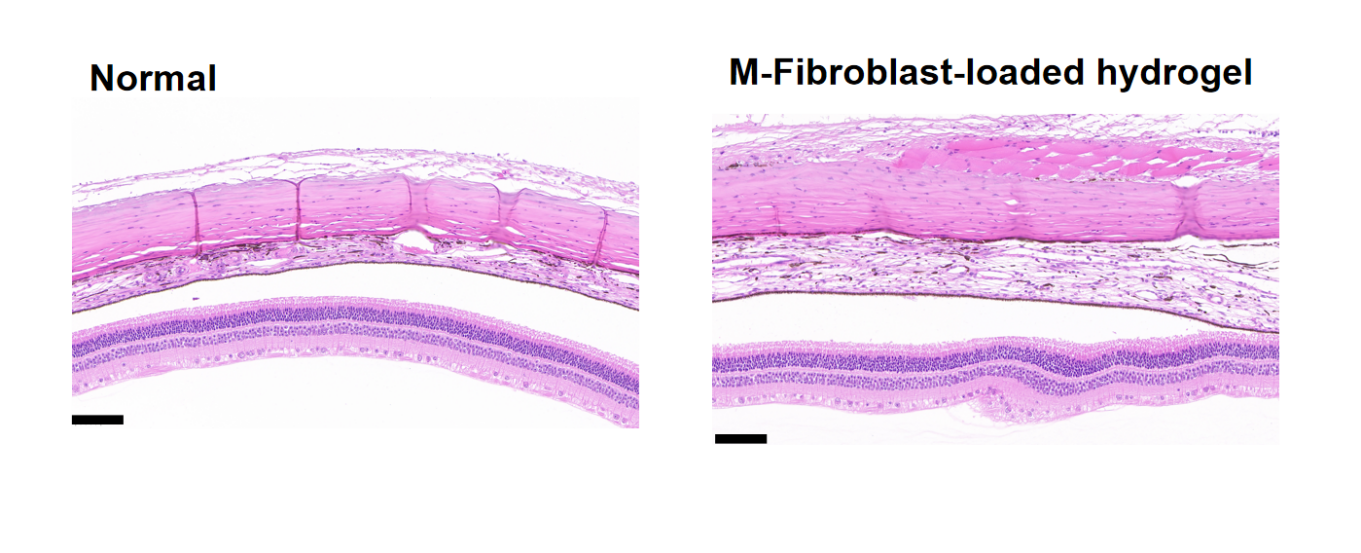


**Figure S6.** H&E staining of sclera-choroid-retina at week 8 in the normal group and fibroblast-loaded hydrogel treatment group. Bar: 100 μm.


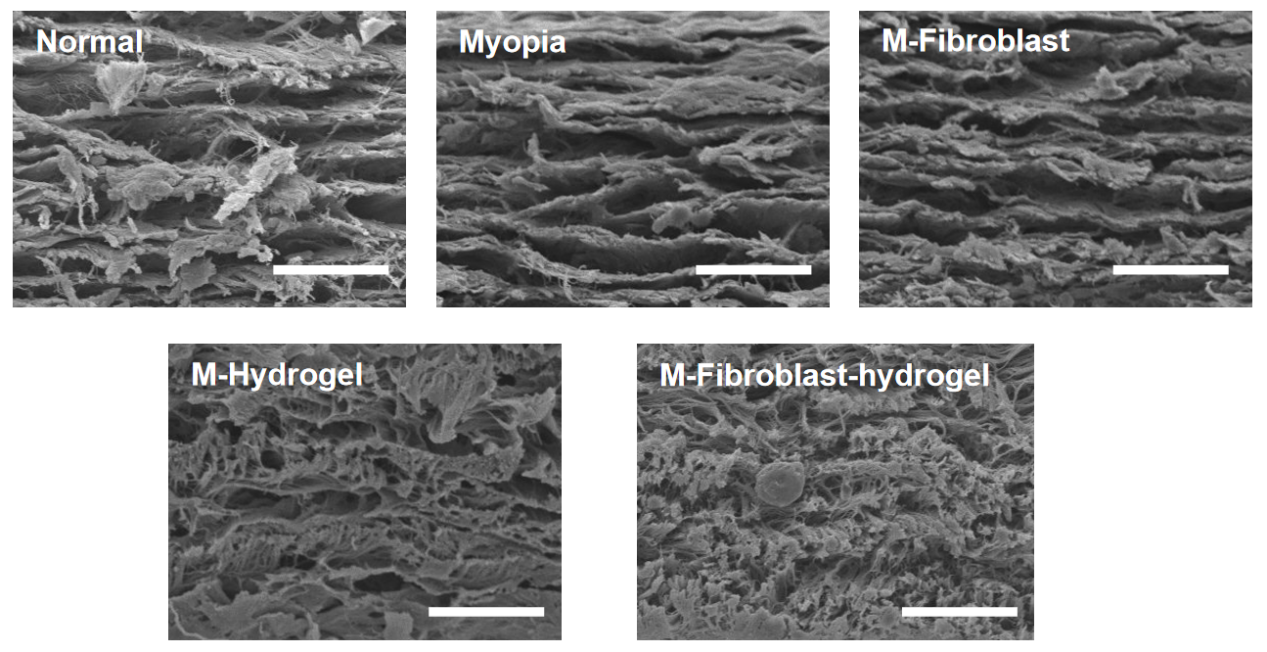


**Figure S7.** High magnification SEM morphologies of sclera in each group at week 4. Bars: 25 μm.


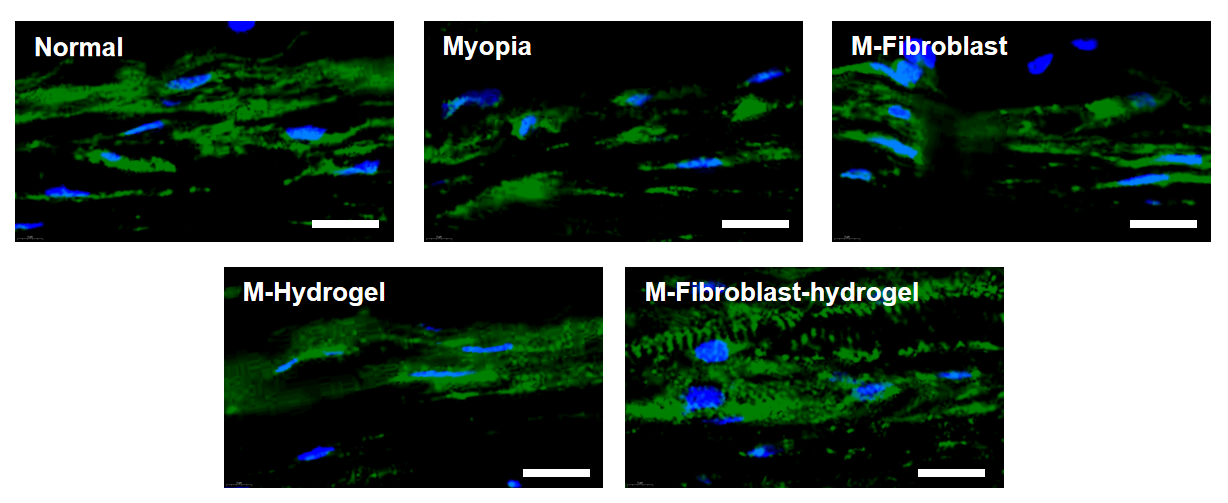


**Figure S8.** High magnification Col Ⅰ (green) / DAPI (blue) immunofluorescence images of the sclera in each group at week 4. Bars: 20 um.

**Table S1.** Change of eye axial length (mm) over 4 weeks in each group.

|  | Normal | Myopia | M-Fibroblast | M-Hydrogel | M-Fibroblast-hydrogel |
| --- | --- | --- | --- | --- | --- |
| Week 0 | 8.17 ± 0.06 | 8.37 ± 0.14 | 8.34 ± 0.10 | 8.35 ± 0.16 | 8.35 ± 0.15 |
| Week 1 | 8.24 ± 0.07 | 8.48 ± 0.10 | 8.44 ± 0.14 | 8.32 ± 0.12 | 8.20 ± 0.09 |
| Week 2 | 8.45 ± 0.11 | 8.56 ± 0.17 | 8.45 ± 0.11 | 8.40 ± 0.10 | 8.15 ± 0.07 |
| Week 3 | 8.53 ± 0.09 | 8.76 ± 0.29 | 8.58 ± 0.11 | 8.57 ± 0.07 | 8.25 ± 0.14 |
| Week 4 | 8.53 ± 0.12 | 8.78 ± 0.21 | 8.72 ± 0.22 | 8.56 ± 0.11 | 8.34 ± 0.15 |
